# Effect of supplementation with ferrous sulfate or iron bis-glycinate chelate on ferritin concentration in Mexican schoolchildren: a randomized controlled trial

**DOI:** 10.1186/1475-2891-13-71

**Published:** 2014-07-15

**Authors:** Ximena Duque, Homero Martinez, Jenny Vilchis-Gil, Eugenia Mendoza, Sergio Flores-Hernández, Segundo Morán, Fabiola Navarro, Victoria Roque-Evangelista, Anayeli Serrano, Robertino M Mera

**Affiliations:** 1 Instituto Mexicano del Seguro Social, Av. Cuauhtemoc No. 330, CP 06725 México, DF, México; 2 RAND Corporation, Santa Monica, California 90407, USA; 3 Hospital Infantil de México Federico Gómez, Dr. Marquez No. 162, CP 06720 México, DF, México; 4 Instituto Nacional de Salud Pública, Universidad No. 658, CP 62100 Cuernavaca, Morelos, Mexico; 5 Vanderbilt University Medical Center, Nashville, TN, USA

**Keywords:** Iron deficiency, Serum ferritin, Ferrous sulfate, Bis-glycinate chelate iron, Schoolchildren

## Abstract

**Background:**

Iron deficiency is one of the most common nutritional deficiencies worldwide. It is more prevalent when iron requirements are increased during pregnancy and during growth spurts of infancy and adolescence. The last stage in the process of iron depletion is characterized by a decrease in hemoglobin concentration, resulting in iron deficiency anemia. Iron deficiency, even before it is clinically identified as anemia, compromises the immune response, physical capacity for work, and intellectual functions such as attention level. Therefore, interventions addressing iron deficiency should be based on prevention rather than on treatment of anemia. The aim of this study was to compare short- and medium-term effects on ferritin concentration of daily supplementation with ferrous sulfate or iron bis-glycinate chelate in schoolchildren with iron deficiency but without anemia.

**Methods:**

Two hundred schoolchildren from public boarding schools in Mexico City who had low iron stores as assessed by serum ferritin concentration but without anemia were randomly assigned to a daily supplement of 30 mg/day of elemental iron as ferrous sulfate or iron bis-glycinate chelate for 12 weeks. Iron status was evaluated at baseline, one week post-supplementation (short term), and 6 months (medium term) after supplementation.

**Results:**

Ferritin concentration increased significantly between baseline and post-supplementation as well as between baseline and 6 months after supplementation. One week post-supplementation no difference was found in ferritin concentration between iron compounds, but 6 months after supplementation ferritin concentration was higher in the group that received bis-glycinate chelate iron. However, there is no difference in the odds for low iron storage between 6 months after supplementation versus the odds after supplementation; nor were these odds different by type of supplement. Hemoglobin concentration did not change significantly in either group after supplementation.

**Conclusions:**

Supplementing with 30 mg/d of elementary iron, either as ferrous sulfate or iron bis-glycinate chelate for 90 days, showed positive effects on increasing ferritin concentration in schoolchildren with low iron stores, and this effect persisted 6 months after supplementation.

## Background

Iron deficiency is one of the most common nutritional deficiencies worldwide. It is more common when iron requirements are increased due to the growth spurts of infancy and adolescence, as well as during pregnancy, when the fetus derives all its iron stores from the mother. The last stage in the process of iron depletion is characterized by a decrease in hemoglobin concentration, resulting in iron deficiency anemia [1,2]. Anemia is the result of a negative iron balance that may be due to any or a combination of the following causes: inadequate iron intake, impaired or low iron absorption, poor iron utilization, or increased iron losses, such as chronic blood loss. In addition to iron deficiency, mineral and vitamin insufficiencies (e.g., folate, vitamin B12, and vitamin A), chronic inflammation, parasite infestation, and hemoglobin (Hb), hereditary disorders can also cause anemia [1,3,4].

Iron homeostasis results from a complex set of events that start by absorption of iron by the intestinal cells, its transport into the cell, and its further release into the blood stream, where it is transported by means of carrier proteins (i.e., transferrin), stored in different body stores, mainly bone marrow, liver, and spleen—known as ferritin complexes—and eventually used in red cell formation by bone marrow. Therefore, different hematological markers—the most common of which include ferritin, serum transferrin, serum transferrin receptors, total serum iron, number and morphology of red blood cells, and Hb concentration—may be used to assess iron status. Given that Hb depletion represents the last stage in iron deficiency, Hb concentration is widely used to diagnose anemia, while serum ferritin is commonly used as indicator of iron status in populations [1,5].

Iron deficiency, even before clinically identified as anemia, compromises the immune response, physical capacity for work, and intellectual functions such as attention level. This is why children are particularly vulnerable to iron deficiency and/or anemia, as they are in a stage of rapid growth. Therefore, interventions addressing iron deficiency should be based on prevention rather than on treatment of anemia [6-8].

The bioavailability and iron absorption from the daily diet are influenced by the type and quantity of iron present in food as well as by the presence of inhibitors and promoters of iron absorption in the diet and the individual’s iron status [8]. It has been estimated that one-fifth of the population worldwide has an iron-deficient diet and that 46% of children between 5 and 14 years are anemic [1,2,9]. The majority of these children live in developing countries. In addition, 56% of pregnant women in developing countries are anemic. Although the precise estimate of what percentage of anemia is due to iron deficiency, parasitical infections or other deficiencies in vitamins, minerals, or nutritious food is not known; it is estimated that 50% of anemia is due to iron deficiency [1,2,9].

According to the 2006 National Health and Nutrition Survey in Mexico (relevant to the time in which the study was conducted), the prevalence of low iron stores in 5–11-year-old children nationwide was 13% [10]. In Mexico City, the prevalence of iron deficiency in children 5–11 years old was 4.5%, with a 95% confidence interval (95% CI) between 2.3 and 8.6, and 11% (95% CI: 7.4 – 14.5) in adolescents 12–14 years old [11]. The prevalence of anemia according to the 2012 National Health and Nutrition Survey in Mexico was 10.1% (95% CI: 9.3 – 10.9) in children 5–11 years old [12]. In school-aged children, the World Health Organization recommends doses of 30 mg/day of iron and 250 mg/day of folic acid for 3 months to prevent iron deficiency anemia [1,8]. In age 0–13 years, the upper tolerable limit recommended by the Institute of Medicine in the United States is 40 mg of elemental iron per day [13]. A recent Cochrane systematic review of iron supplementation in children addressed the effects of intermittent oral iron supplementation versus no supplementation (placebo) in children under 12 years of age found that iron supplementation improves hematological outcomes. The risk ratio (RR) of presenting anemia was 0.51 (95% CI: 0.37 – 0.72), while the RR of presenting iron deficiency was 0.24 (95% CI: 0.06 – 0.91), and iron status assess by serum ferritin showed higher mean levels for those receiving supplements compared to those receiving no treatment [1]. It should be noted, however, that most of the 33 studies included in this review were conducted in settings with a high prevalence of anemia.

Despite the wide range of pharmaceutical products available to treat anemia, there have been few studies about school-age children concerning the effect of preventive supplementation on nutritional iron status. Further, few studies have compared the relative effectiveness of different iron compounds. Ferrous sulfate is the most commonly used iron compound in supplementation programs because of its efficiency and low cost [1,14,15]. Iron bis-glycinate chelate has been proposed as an alternative to ferrous sulfate because of its increased bioavailability. Due to its chemical composition, with a ferrous cation coupled to two glycine molecules, it does not form insoluble compounds with substances like phytates, oxalates, and tannins present in high quantity in cereal-based diets, which have a high content of iron absorption inhibitors [16-19]. Furthermore, this type of iron has caused less collateral effects with high doses of 60 mg/d or more of elementary iron [20]. Therefore, in equal doses, iron bis-glycinate chelate would be expected to have a larger effect on body iron status compared to ferrous sulfate [19,21-23]. However, most of the studies using this form of iron relate to food enrichment interventions [19,21-23].

Given that iron supplementation studies have clearly shown positive effects on treatment of anemia [1,8,9,14,15], it is important to increase our knowledge on the prevention of iron deficiency anemia. The objective of this study was to compare the effect on serum ferritin concentration of daily supplementation with 30 mg of elemental iron as ferrous sulfate versus iron bis-glycinate chelate in schoolchildren with iron deficiency but without anemia.

## Methods

A randomized, double-blind clinical trial was conducted between June 2005 and March 2008, enrolling children aged 5–13 years old from three elementary schools in Mexico City. This study was carried out with children from low-income families. All of them attended public boarding schools where the children remain five days per week and go home on weekends and vacations. Diets in participating boarding schools are very similar, as the menus and food delivered are run from a central facility and standardized across participating schools.

A sample size was calculated following the rationale for a bioequivalence study [24]. Based on previous information, the probability of success with the administration of iron sulfate was calculated as 0.81; while success with iron bis-glycinate was calculated as 0.86, we set the maximum allowable difference at no more than 0.1. The calculated sample size rendered 75 children per group, to which we added an additional 20% for possible attrition, for a final sample size of 90 children per group.

Hb and ferritin concentrations were measured to screen for potential participants. Children with normal hemoglobin concentration but with ferritin lower than the cut-off were considered to have low iron stores but no anemia and were included in the study. The following cut-offs were applied: serum ferritin <12 μg/l and Hb adjusted for altitude ≥120 g/l for children 12 years or older and ≥115 g/l for children less than 12 years [8,25]. Children with Hb and ferritin lower than the specified cut-off were considered as having iron deficiency anemia; they received treatment with the standard oral iron treatment but were not included in the study.

### Study intervention

Children with iron deficiency without anemia were randomly assigned to daily supplements with ferrous sulfate or bis-glycinate chelate iron (30 mg of elemental iron plus 100 μg of folic acid) for 90 days. Computer-generated random numbers (http://www.randomization.com) were used to assign each child with iron deficiency to either supplementation group. Supplement pills were identical in size, form, and color, and were coded to preserve blinded assignment. Supplementation was administered Monday through Friday by trained project staff. The person responsible for administering the supplement kept a schedule and a registry of which child received the supplement, according to the random assignment. Compliance was assessed by means of the daily registration of supplement administration at school, according to the schedule kept by project staff responsible for its administration. For non-school days (i.e., weekends, holidays, or days with no school attendance), parents were in charge of keeping the schedule and writing down information about the assigned supplement pill’s administration. This same schedule included questions related to potential side effects. All participants received a single 400 mg albendazol dose at the beginning of the study as anti-helminthic prophylaxis.

### Biochemical determinations

A venous blood sample was taken by skilled personnel on three occasions to determine serum ferritin and Hb concentrations. The samples were taken at baseline, a week after completing iron supplementation, and 6 months after treatment completion (6 months post-treatment). The sample was taken only if the child did not have a cold or gastrointestinal infection symptoms (pain, temperature, cough, vomiting, or diarrhea) the week before. If symptoms had been present, blood samples were taken at a later date. Blood cytology samples were analyzed by cellular counter (Beckman Coulter, Fullerton, CA). Ferritin was determined by immune-radiometric assay (Inmunotech SA, Marseille, France).

### Anthropometry and diet

All participants were weighed and measured using standard procedures as suggested by Lohman [26]. Body mass index (weight in kg/height in m^2^) was calculated and children were categorized by age and gender as having normal weight, being overweight, or being obese [27]. The height-for-age *z* score was calculated and children were classified as showing normal nutritional status (*z* score ≥ −1) or a slight-to-moderate malnutrition status (*z* score < −1) [28].

Semi-quantitative 24-hour recall questionnaires were used to evaluate the children’s nutrient intake. Standard portions of the different foods offered at school were weighed and the ingredients and quantities were recorded. Each child was asked how much food he or she had eaten using a 3-option scale: everything, nothing, or about half of what was served. Any food eaten by the children other than what was offered in school was recorded. The Mexican Table for Food Equivalence and the Mexican Food Composition Table [29,30] were used to calculate the energy and nutrient (protein, carbohydrate, lipids, iron, and vitamins) intake.

### Sociodemographic characteristics

Sociodemographic information including age, gender, number of occupants in the dwelling, and parents’ education and occupation was collected through a questionnaire given to schoolchildren’s parents or tutors.

### Ethical approval

This study was conducted according to the guidelines of the Declaration of Helsinki and was approved by the Research and Ethics Committees of the Mexican Institute of Social Security in Mexico City. Written consent was obtained from the participants’ parents or tutors, and written assent was requested from children older than 7 years.

### Statistical analysis

Descriptive statistics were used to describe the baseline status of the study population. Variables related to iron nutritional status, Hb, and ferritin concentration were analyzed using continuous and categorical scales.

The effect of supplementation was evaluated within and between groups, one week after supplementation and 6 months after supplementation. Student’s *t*-test was used to evaluate the difference in Hb and ferritin concentration between groups. Pearson’s *Χ*^2^ test was used to analyze changes in the classification of nutritional iron status between groups.

Multivariate analyses to evaluate effects of supplementation on hemoglobin and ferritin concentration during the study with the two iron compounds were carried out using mixed-effects linear regression models. The models were adjusted for fix variable gender and for age as a variable time dependent. Possible interaction between the iron compound and the time of assessment of the outcome variables Hb and ferritin concentration were evaluated. As time of assessment and iron compound interaction was statistical significance in the model to ferritin concentration, the mean of ferritin by iron compound and study stage was described using margins analyses [31].

Mixed-effects logistic regression model was used to evaluate the risk of having iron deficiency after supplementation by iron supplementation group adjusting by age, sex, ferritin, and hemoglobin concentration at baseline.

Values of *p* < 0.05 were considered statistically significant. All data analyses were carried out using Stata 12 SE (Stata Corp., College Station, TX).

## Results

A total of 1,020 children were screened, and 200 (19.6%) who showed low iron stores without anemia were included in this study. Children were randomly allocated to two treatment groups: 101 children received iron bis-glycinate chelate and 99 children received ferrous sulfate.

**Figure 1:**
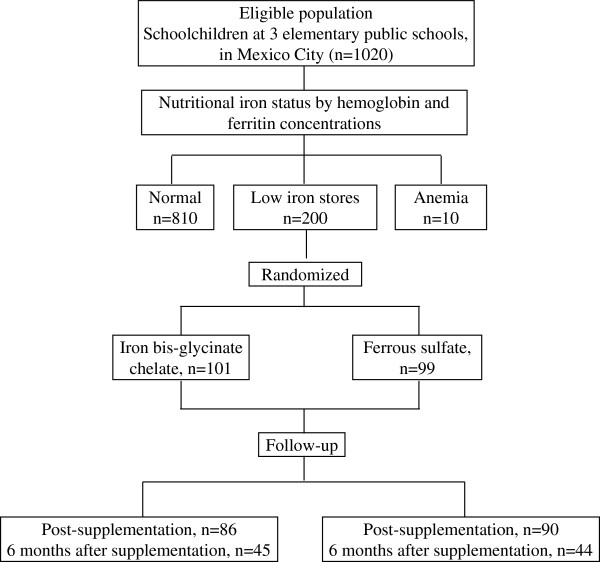
**Study population.** Flow of participants through the trial.

A total of 176 children (88%) who received iron supplementation were available for the one-week post-supplementation assessment of iron status. There were missing values for 14.9% of children who received iron bis-glycinate chelate, and 9.1% for children who received ferrous sulfate. Six months after supplementation, there were samples available for 89 children (44.5% of the total intervention population). Losses to follow-up at 6 months after supplementation were 55.4% for the group supplemented with bis-glycinate chelate and 55.6% for the group supplemented with ferrous sulfate (Figure 1). There were no statistically significant differences between the baseline characteristics of children who completed the study and those who were lost to follow-up, except that children who had missing values 6 months after supplementation were older than children who completed the study. The age difference was statistically significant for the iron bis-glycinate chelate group (8.7 ± 1.9 vs. 9.7 ± 2.1, *p* = 0.018). Fear and unpleasantness related to taking blood samples were the most common reasons for children not to complete the study.

There were no cases in which supplementation had to be discontinued due to undesirable side effects or gastrointestinal discomfort. Twenty-two children (10.4%) had one of the following side effects for one or more days: diarrhea (*n* = 3), abdominal pain (*n* = 15), nausea (*n* = 9), constipation (*n* = 2), or metallic taste (*n* = 11). There were no significant differences between the groups in the presentation of side effects potentially associated to iron supplementation.

The mean energy intake, after adjusting for age and sex, was 9188 ± 468 KJ/d. The calorie distribution value of macronutrient intake was 15.3% from proteins, 56.3% from carbohydrates, and 27.8% from lipids, and the mean iron consumption was 11.7 ± 0.7 mg/d. There were no significant differences in the mean intake of micronutrients or iron between supplemented groups.

### Study population characteristics

The mean age of children with low iron stores was 9.3 ± 1.9 years. The mean of ferritin was 8.7 ± 2.1 μg/l. The mean of Hb was 140 ± 9.1 g/l. There were no significant statistical differences in the sociodemographic or nutritional characteristics between supplementation groups (Table 1).

**Table 1:** Characteristics of study population according to supplementation group

**Characteristics/Supplement compound**	**All *N *** **=200**		**Bis-glycinate chelate iron *n*** **= 101**		**Ferrous sulfate *n*** **= 99**		***P *** **value**^**a**^
	***n***	**%**	***n***	**%**	***n***	**%**	
Sex							
Male	91	45.5	42	41.6	49	49.5	
Female	109	54.5	59	58.4	50	50.5	0.261
Age (years)^b^	9.3	1.9	9.3	2.1	9.4	1.7	0.728
Height for age^c^							
Normal	136	69.1	72	72.7	64	66.0	
Low or moderate malnutrition	60	30.6	27	27.3	33	34.0	0.305
Body mass index^d^							
Normal	147	73.0	70	70.7	73	75.3	
Overweight or obese	53	27.0	29	29.3	24	24.7	0.473
Mother’s schooling							
High school or more	60	32.3	30	31.9	30	32.6	
Middle school or less	126	67.7	64	68.1	62	67.4	0.919
Hemoglobin concentration (g/l)^b^	140.9	9.1	141.6	10.3	140.2	7.6	0.273
Ferritin concentration (μg/l)^b^	8.7	2.1	8.7	2.1	8.9	2.1	0.548

^a^Pearson’s *X*^2^ test.

^b^Mean and standard deviation. Student’s *t* test.

^c^WHO’s reference population, 2007 [28].

^d^Cole et al. Cut-off point by age and sex [27].

### Descriptive results: serum ferritin and hemoglobin concentration

During the follow-up, there was a statistically significant positive effect of supplementation on ferritin concentration and on the classification of iron status. At baseline, all schoolchildren had low iron storages as evaluated by ferritin concentration; one week after supplementation and 6 months post-supplementation, only 10% of children had low iron storages (Table 2).

**Table 2:** Comparison of nutritional iron status at baseline and during the follow-up by iron compound supplement

	**Baseline**					**Post-supplementation**					**6 months after supplementation**				
**Iron Status/Supplement compound**	**Bis-glycinate chelate *n*** **= 101**		**Ferrous sulfate *n*** **= 99**		***P *** **value**	**Bis-glycinate chelate *n*** **= 86**		**Ferrous sulfate *n*** **= 90**		***P *** **value**	**Bis-glycinate chelate *n*** **= 45**		**Ferrous sulfate *n*** **= 44**		***P *** **value**
Hemoglobin concentration (g/l)^a^	141.6		140.2			141.7		140.1			141.7		139.8		
Standard deviation	10.3		7.6		0.273^b^	8.2		7.5		0.175^b^	7.3		6.9		0.205^b^
Ferritin concentration (μg/l)^a^	8.7		8.9			29.6		28.9			31.0		25.2		
Standard deviation	2.1		2.1		0.548^b^	15.1		13.1		0.728^b^	18.7		12.5		0.090^b^
Iron nutritional status^c^															
Low iron stores	101	100	99	100		9	10.5	6	6.7		5	11.1	4	9.1	
Normal	0	0.0	0	0.0	--	77	89.5	84	93.3	0.367^d^	40	88.9	40	90.9	0.752^d^

^a^Mean and standard deviation.

^b^Student’s *t* test.

^c^Low iron stores = Serum ferritin <12 μg/l.

^d^Pearson’s *Χ*^2^ test.

### Effects on ferritin and hemoglobin concentration and on the risk of having iron deficiency

**Figure 2:**
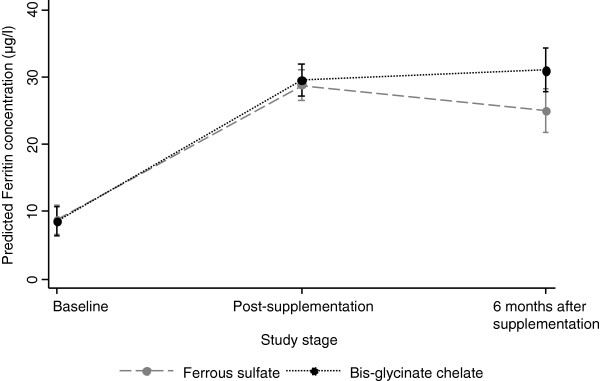
**Predicted ferritin concentration (μg/l) by iron compound and time of evaluation.** Results are based on the model shown in Table 3.

Multivariate analysis on the effect of supplementation on ferritin concentration after adjusting by age at the time of each biochemical determination and by sex showed the following: 1: Compared to baseline ferritin concentration, the children had a change of 20.16 μg/l one week post-supplementation and a change of 16.43 μg/l 6 months after supplementation. 2: The effect was statistically different between iron compounds only at 6 months after supplementation. The group that received bis-glycinate chelate iron showed 6.04 μg/l higher ferritin concentration in comparison to the group with ferrous sulfate supplementation, *p* = 0.028 (Table 3, Figure 2). The adjusted ferritin concentration one week post-supplementation, based on the model shown in Table 3, was estimated to be 28.9 μg/l (95% CI: 26.6 – 31.2) and 6 months after supplementation of 25.2 μg/l (95% CI: 21.9 – 28.5) in the group that received ferrous sulfate, and of 29.6 μg/l (95% CI: 27.3 – 32.0) and 31.1 μg/l (95% CI: 27.8 – 34.3), respectively, in the group that received bis-glycinate chelate (Figure 2). No significant effect was observed in Hb concentration (Table 4).

**Table 3:** Effects on mean concentration of ferritin according to iron compound and time of evaluation

**Supplement and time of evaluation**	**Change in mean of ferritin (μg/l)**	**95% CI**	***P *** **value**
**Supplement**			
Ferrous sulfate	Ref.		
Bis-glycinate chelate	−0.18	−3.26 – 2.91	0.909
**Time of evaluation**			
Baseline	Ref.		
One week post-supplementation	20.16	17.11 – 23.22	0.001
6 months after supplementation	16.43	12.57 – 20.28	0.001
**Additional increment in the group supplemented with Bis-glycinate chelate**^**a**^			
At baseline	Ref.		
One week post-supplementation	0.87	−3.45 – 5.19	0.694
6 months after supplementation	6.04	0.64 – 11.43	0.028

Mixed-effects linear regression model with random intercept, adjusted by age and sex. Number of observations: 465, number of groups: 200, observations per group: 2.3 (1–3).

Ref. = Reference value.

^a^Interaction between study stage and iron compound (bis-glycinate chelate vs. ferrous sulfate by time of evaluation).

**Table 4:** Effects on mean concentration of hemoglobin (g/l) according to iron compound and time of evaluation

**Supplement and time of evaluation**	**Change in mean Hb**	**95% CI**	***P *** **value**
**Supplement**			
Ferrous sulfate	Reference		
Bis-glycinate chelate	1.71	−0.20 – 3.63	0.080
**Time of evaluation**			
Baseline	Reference		
One week post-supplementation	−0.11	−1.36 – 1.13	0.859
6 months after supplementation	−0.46	−2.08 – 1.16	0.578

Mixed effects linear regression model with random intercept, adjusted by sex and age. Number of observations: 469, number of groups: 200, observations per group: 2.3 (1–3).

There were no differences between the odds of having low iron storages either in the group with iron bis-glycinate chelate versus the group that received ferrous sulfate, OR 1.40 (CI 95%, 0.58 – 3.42) either at 6 months post-supplementation versus one week post-supplementation, OR 1.40 (CI 95%, 0.56 – 3.49), after adjustment for age, sex, ferritin, and hemoglobin concentration at baseline (Table 5).

**Table 5:** Association between iron compound at time of evaluation and low iron storages

**Supplement and time of evaluation**	**OR**	**95% CI**	***P *** **value**
**Supplement**			
Ferrous sulfate	Ref.		
Bis-glycinate chelate	1.40	0.58 – 3.42	0.452
**Time of Evaluation**			
One week post-supplementation	Ref.		
6 months after supplementation	1.40	0.56 – 3.49	0.465

Ref. = Reference value.

Mixed effects logistic regression model with random intercept, adjusted by sex, age; ferritin and hemoglobin concentration at baseline. Number of observations: 265, number of groups: 180, observations per group: 1.5 (1–2).

## Discussion

Our results showed that 90 days of supplementation with 30 mg/day of elemental iron as either ferrous sulfate or iron bis-glycinate chelate had a positive effect on raising ferritin concentration in school-age children with low iron stores. This effect was seen one week post-supplementation and was still present 6 months after supplementation. Furthermore, the bis-glycinate chelate compound was more efficient for maintaining higher ferritin concentration 6 moths after supplementation than ferrous sulfate. Persistence of the observed effect for this long period after supplementation may be explained by the effect of a diet that may provide enough bioavailable iron to maintain iron status once the initial deficit was overcome. Actually, the effect of iron bis-glycinate chelate may be larger where individuals consume a typical Mexican diet (as do families with low incomes, such as the ones included in this study), which includes phytic acid from cereals (i.e., maize) and legumes (i.e., beans). This compound binds iron, reducing its bioavailability for intestinal absorption.

In a longitudinal study about iron deficiency due to consumption of low bioavailable iron diet in Africa by schoolchildren with normal iron status after a fortification intervention, after 15 months the authors found a mean change in total body iron stores of −142 mg, and estimated a 2% mean iron absorption of dietary iron. Hemoglobin decreased by 12 g/l, and 75% of the cohort had deficit in tissue iron. The authors concluded that low iron bioavailability from a legume- and cereal-based diet is a cause of iron deficiency anemia in children in rural Africa [32]. In our study, there were no differences in the odds of having low iron storages between the two iron compounds at either evaluation period. This could mean that the higher ferritin concentration at 6 months post-supplementation in the bis-glycinate chelate group versus ferrous sulfate group had no clinical implication.

Our findings concurred with other studies carried out on pregnant women, adolescents, school-age children, and preschool children in which both ferrous sulfate and iron bis-glycinate chelate were found to be equally effective [20,33]; however, in those studies, the effects 6 months post-supplementation had not been evaluated. Results of a systematic review and meta-analysis on iron supplementation on children, that included 32 studies including 7,089 children aged 5–12 years; 31 of these studies were conducted in lower- or middle-income countries [9] and showed that iron supplementation improves hematologic and non-hematologic outcomes. Iron supplementation decreased the prevalence of anemia by about 50% and reduced the prevalence of iron deficiency by 79%; there was also evidence of a benefit on cognitive performance in children with anemia at baseline [9]. The increase in hemoglobin or ferritin concentrations is related to iron status at baseline, dose of iron, and length of time of supplementation [3,34,35]. However, most studies have focused on treating anemia more than on treating the iron deficiency to prevent iron deficiency anemia.

In a supplementation study carried out in Brazil that compared administration of ferrous sulfate (40 mg of iron per week) versus iron bis-glycinate chelate (3.8 mg per week as bis-glycinate chelate-enriched cookies) for 8 weeks on schoolchildren with anemia, the authors reported a significant increase in Hb concentration but no significant difference in the inter-group comparison. No effect was observed on serum ferritin for either intervention, but children with depleted iron stores (ferritin <15 ng/mL) at the beginning of the intervention showed increased serum ferritin concentration, although no difference between treatments was observed [33].

Ribeiro and Sigulem supplemented anemic children between 6 and 36 months with 5 mg/kg/d of iron as bis-glycinate chelate [36] and found a significant increase in the Hb concentration; however, ferritin concentration was not measured. Pineda and Ashmead supplemented children with anemia and malnutrition between 6 to 36 months of age with ferrous sulfate versus iron bis-glycinate chelate offering 5 mg/kg/d for 28 days. This study showed a significant increase in Hb and ferritin concentration in both supplemented groups. The difference in Hb concentration between the two groups post-intervention was not statistically significant; however, the difference in the serum ferritin concentration was significantly higher in the group that received iron bis-glycinate chelate [37]. In summary, these studies show a positive effect of supplementation with ferrous sulfate and of supplementation or food fortification with ferrous bis-glycinate chelate on treating anemia, but we found no published study that focused on preventing iron deficiency anemia in schoolchildren population that included the bis-glycinate chelate iron compound.

As mentioned, iron bis-glycinate chelate has been used for food enrichment and for supplementation. Due to its chemical structure (consisting of a covalently bounded iron molecule to an organic ligand, in this case glycine), this form of iron can be partially resistant to the action of enzymes and to the binding action of substances naturally present in food such as metals, dietary fiber, phytates, and phenols, with which the iron can form insoluble compounds. In addition, because of the amino acid bonding, there is less direct exposure of iron to the gastrointestinal mucosa cells; this can reduce local toxicity and side effects attributable to iron compounds present in the intestinal lumen, like abdominal pain [18,19,33,37,38]. In various supplementation studies, correlations have been noted between the doses of iron given and/or the chemical formula used and side effects. The most common side effects associated with iron supplementation affect the gastrointestinal tract, manifesting as constipation, nausea, diarrhea, and vomiting [1,3,36,38].

Coplin et al. compared the tolerability of ferrous sulfate and iron bis-glycinate chelate in a study. They found that both ferrous sulfate and iron bis-glycinate chelate had equivalent therapeutic efficiency at a similar dosage (50 mg), but the side effects were greater (37% vs. 21%) in the group that received ferrous sulfate [20]. Gastrointestinal complaints and symptoms as constipation, nausea, vomiting, and diarrhea have been observed commonly in women consuming high doses of supplemental iron. The frequency and intensity of these complains are related to the amount of elemental iron released in the stomach [1,39]. In malaria areas, additional iron may exacerbate malaria infection. For theses reasons, iron supplementation programs should be supervised and planned according to target the population [1].

In a systematic review, Low et al. evaluated the benefits and safety of daily iron supplementation in school-aged children. There was no difference in the frequency of children with gastrointestinal upset, constipation, vomiting, or diarrhea between children who received iron supplementation and children in the placebo group. The authors concluded that iron supplements appeared to be well tolerated, but the safety dates were limited: only 6 of 32 studies reported safety outcomes [9]. In our study, there were few gastrointestinal side effects with either treatment, so there was no need to suspend supplementation. Moreover, there were no differences in the observation of gastrointestinal side effects between the two supplementation groups. This lack of significant side effects may have been due to the low dose of iron given (30 mg/d).

We recognize some caveats of our study. In terms of design, a more comprehensive description of iron status in the body would include other indicators of iron status, such as serum transferrin, serum transferring receptors, and total serum iron. Likewise, indicators of infection as C reactive protein and α1-glycoprotein have been evaluated in some studies on iron supplementation. However, serum ferritin has been considered a good indicator of iron storage and is useful in evaluating interventions response at a population level [2].

While serum concentrations of this protein can be increased during inflammatory and infectious processes as well as in response to trauma, children participating in our study received anti-helminthic prophylaxis at the beginning of the study, and the blood sample was obtained only if the children did not have a cold, fever, or any other common symptom of infection, so we consider that the use of serum ferritin in this study was a good indicator of iron storage as well as a good indicator of intervention response at the population level, as has been suggested [5]. In terms of implementation, the main constraint of our study was related to the high attrition rate to effect evaluation at 6 months after supplementation. However, the only difference between participants who completed the study and the ones missing data during follow-up was age, and the results of our multivariate analyses were adjusted by age as soon as each evaluation of iron status was done. Therefore, we do not think that this attrition reflected a selection bias that could substantially bias our results.

## Conclusions

The results of our study showed that a low iron dose of 30 mg/day for 90 days with either ferrous sulfate or iron bis-glycinate chelate significantly increased serum ferritin concentration in schoolchildren with low iron stores but no anemia and had negligible side effects. The effect on increased iron status was sustained up to 6 months after supplementation, rendering both treatments as safe and effective. These results support the preventive effectiveness of this low-dose iron intervention to increase serum ferritin, which will help prevent iron deficiency anemia and may be relevant where iron deficiency is a public health concern and where a preventive daily low dosage of iron supplementation can be used to help the school-age population maintain an adequate nutritional iron status.

We recognize upfront, however, that iron supplementation is no substitute for a balanced diet and that in areas where parasitic infections are prevalent, these should be treated as part of a comprehensive approach to improve iron status. Furthermore, where malaria is endemic, we would caution against extended use of iron supplementation, even in small doses, until safety studies have been conducted. In these areas, the World Health Organization recommends to first treat the infection, and then to provide iron supplementation. In summary, while our results support that low daily doses of ferrous sulfate or bis-glycinate chelate iron can be utilized to prevent iron deficiency anemia in schoolchildren with iron deficiency, prevention and control of anemia are complex and, depending on the setting, may require the implementation of a comprehensive set of control measures [40].

## Competing interests

The authors declare that they have no competing interests.

## Authors’ contributions

XD designed the study, analyzed data, and drafted the manuscript. HM critically revised the manuscript for important intellectual content and participated in interpretation of results. JV designed the study and managed the data base. RM coordinated the statistical analyses and participated in the interpretation of results. SF participated in the study design and statistical analyses. SM participated in the study design and results interpretation. EM was in charge of lab analyses and participated in the interpretation of results. FN, VR, and AS conducted the data collection, supplementation and collaborated in the interpretation of results. All authors read and approved the final manuscript.
